# Sustainable Postharvest Innovations for Fruits and Vegetables: A Comprehensive Review

**DOI:** 10.3390/foods14244334

**Published:** 2025-12-16

**Authors:** Valeria Rizzo

**Affiliations:** Department of Biosciences and Technology for Food Agriculture and Environment, University of Teramo, Via R. Balzarini 1, 64100 Teramo, Italy; vrizzo@unite.it

**Keywords:** circular economy, processing innovations, storage technologies, postharvest losses, food waste, shelf-life extension, nanocoatings, bioactive compounds, quality preservation

## Abstract

The global food industry is undergoing a critical shift toward sustainability, driven by high postharvest losses—reaching up to 40% for fruits and vegetables—and the need to reduce environmental impact. Sustainable postharvest innovations focus on improving quality, extending shelf life, and minimizing waste through eco-efficient technologies. Advances in non-thermal and minimal processing, including ultrasound, pulsed electric fields, and edible coatings, support nutrient preservation and food safety while reducing energy consumption. Although integrated postharvest technologies can reduce deterioration and microbial spoilage by 70–92%, significant challenges remain, including global losses of 20–40% and the high implementation costs of certain nanostructured materials. Simultaneously, eco-friendly packaging solutions based on biodegradable biopolymers and bio-composites are replacing petroleum-based plastics and enabling intelligent systems capable of monitoring freshness and detecting spoilage. Energy-efficient storage, smart sensors, and optimized cold-chain logistics further contribute to product integrity across distribution networks. In parallel, the circular bioeconomy promotes the valorization of agro-food by-products through the recovery of bioactive compounds with antioxidant and anti-inflammatory benefits. Together, these integrated strategies represent a promising pathway toward reducing postharvest losses, supporting food security, and building a resilient, environmentally responsible fresh produce system.

## 1. Introduction

Postharvest losses of fruits and vegetables represent one of the most pressing challenges for global food security and environmental sustainability. Due to their high perishability, improper handling, transportation and storage frequently lead to significant waste, with global losses ranging between 20% and 40% [1].

This corresponds to over one-third of food intended for human consumption [2], creating significant impacts on economic stability, environmental health, and resource efficiency. Such losses are particularly severe in low- and middle-income countries (LMICs), where limited infrastructure and inadequate cold-chain systems often push losses above 30% [3,4].

From the standpoint of sustainability, a food value chain is considered sustainable when it ensures social and economic development while preserving planetary resources for future generations [5]. Reducing food loss and waste (FLW) is therefore crucial to achieving the Sustainable Development Goals (SDGs), especially SDG 12.3, which targets the halving of global FLW; SDG 9, which encourages investment in innovation and resilient infrastructures; and SDG 13, centered on climate action [6,7]. Given that global food demand is projected to rise by 2050, the adoption of adequate postharvest strategies is not optional but essential for nutritional security [5,7].

There is an urgent need to adopt multilevel strategies—including improvements in food handling, education, infrastructure, and research capacity—to enhance environmental sustainability. This urgency is reinforced by global concerns: FAO and EU reports emphasize that the failure to mitigate and adapt to climate change represents one of the highest global risks (World Economic Forum, 2016) [7,8]. In response, governments worldwide are promoting sustainable economic development by encouraging businesses to reduce waste and energy consumption. Sustainable technologies—applied to products, processes, and services—are increasingly viewed as effective tools to minimize environmental impact through reduced raw-material usage and cleaner production pathways [9], a need further underscored by the fact that approximately 30–40% of fresh produce is lost annually, primarily due to inadequate postharvest handling, storage, and transportation [10].

**Figure 1:**
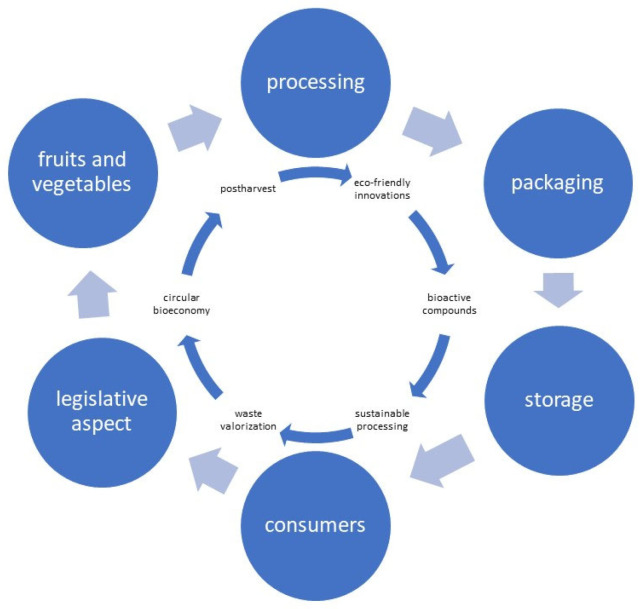
Schematic conceptual framework.

This review aims to evaluate innovative and sustainable strategies for postharvest disease management in fruits and vegetables—encompassing processing techniques, new packaging solutions (e.g., nanotechnology and edible coatings), storage, and legislative aspects, as well as their links to consumer perceptions and economic feasibility—while promoting the dissemination of research on effective and sustainable solutions that address the technical, economic, and regulatory barriers to their implementation, as outlined in the conceptual framework (Figure 1).

**Figure 2:**
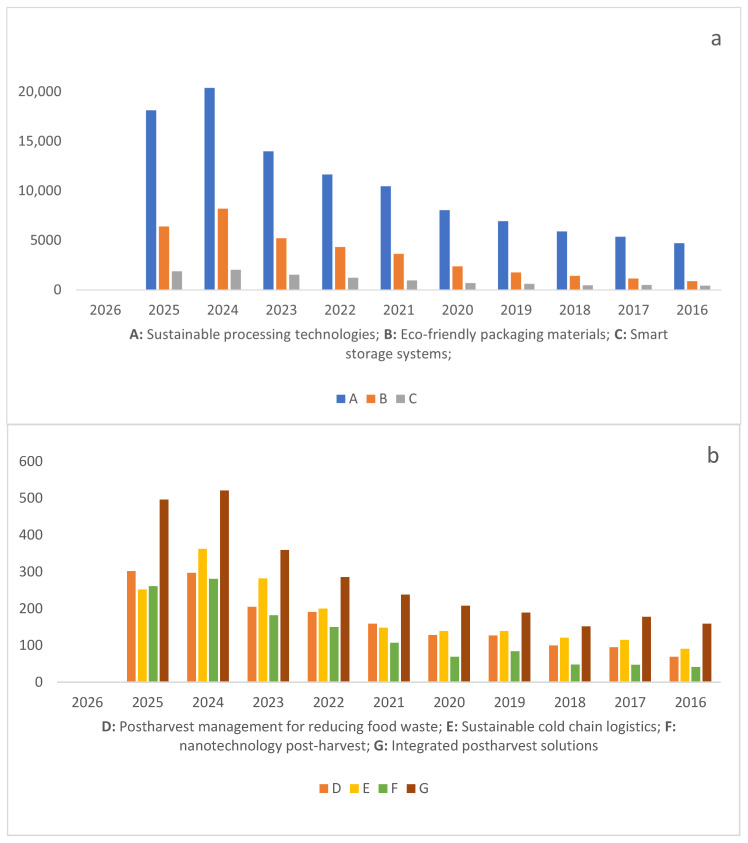
(**a**) A: Sustainable processing technologies; B: Eco-friendly packaging materials; C: Smart storage systems; (**b**) D: Postharvest management for reducing food waste; E: Sustainable cold chain logistics; F: nanotechnology post-harvest; G: Integrated postharvest solutions.

To further frame the relevance of this review, the scientific landscape in this field shows a substantial and growing research effort. Numerous literature reviews have been published across different subtopics, and publication volumes confirm a strong international focus on sustainable postharvest innovations. A search on ScienceDirect (2016–2026), limited to “Agricultural and Biological Sciences”, reveals particularly high output in sustainable processing technologies (105,628 articles) and eco-friendly packaging (35,452 articles) (Figure 2).

The areas with the highest publication volumes include sustainable processing technologies (105,628 articles) and eco-friendly packaging materials (35,452 articles) (Figure 2a). Other crucial areas driving current scientific inquiry are: (Figure 2b)

- Smart storage systems (10,290 articles).
- Integrated postharvest solutions (2787 articles).
- Postharvest management for reducing food waste (1850 articles).
- Sustainable cold chain logistics (1674 articles).
- The applications of nanotechnology post-harvest (1270 articles).

These publication patterns reveal clear trends in current research priorities, particularly the focus on sustainable processing technologies and eco-friendly packaging, which together dominate the scientific output. This emphasis probably reflects growing industry demand for scalable, low-impact processing solutions and the need to reduce environmental burdens across the supply chain. In contrast, the lower volumes in areas such as smart storage systems, integrated postharvest solutions, and sustainable cold-chain logistics highlight emerging niches driven by technological innovation and by the global push to minimize postharvest losses. The expanding interest in nanotechnology applications further underscores a shift toward advanced, high-precision tools aimed at improving food quality, safety, and shelf life.

## 2. Sustainable Postharvest Technologies

### Challenges, Opportunities and Critical Gaps

Postharvest deterioration is driven by physiological, biochemical, microbial, and logistical factors. Traditional mitigation strategies often rely on energy-intensive processes, synthetic chemicals, or non-renewable packaging materials, each carrying environmental and health trade-offs. Therefore, sustainable postharvest innovations must simultaneously address multiple dimensions: improved food safety, reduced environmental footprint, cost-effectiveness, and compliance with increasingly strict regulatory frameworks.

Fruits and vegetables are metabolically active and highly perishable, undergoing quality deterioration due to natural ripening and senescence processes. Their high moisture content and nutritional factors make them particularly susceptible to rapid deterioration and conducive to the growth of spoilage microorganisms [2,4,5]. Postharvest losses are primarily driven by physiological deterioration caused by technical, biological, and environmental factors [11]. Challenges arise because these commodities require prompt and efficient postharvest intervention due to their inherently short shelf life [4].

Inadequate preservation practices result in detrimental economic impacts throughout the supply chain. The magnitude of postharvest loss in the Mediterranean region, for example, in 2010–2015 was estimated to exceed USD 50 billion annually [12]. Losses negatively affect farmers’ earnings, household income, and overall food security [4]. Furthermore, FLW has alarming consequences for micro-nutrient losses, such particularly vitamins A and C, especially in fresh fruit and vegetables [6]. Addressing these inefficiencies is vital, considering the global attribution of $15 billion in losses and a 12% carbon footprint to postharvest inefficiencies [10].

Consumer perceptions are shifting toward greater concern regarding the environmental consequences of packaging materials [13]. This mindset strongly influences the push toward more sustainable alternatives and away from conventional petroleum-based plastics [12,14,15]. Consumers are increasingly demanding minimally processed and preservative-free foods, favoring natural, eco-friendly food solutions and clean-label products [16,17,18]. This trend encourages the adoption of sustainable and innovative disease control methods, rather than traditional chemical methods [1].

Recent advances in non-thermal and minimal processing methods allow quality preservation while reducing energy consumption and chemical use. Techniques such as high-pressure processing, pulsed electric fields, and UV treatments help maintain nutrients, color, and microbial safety. Advanced postharvest physical and chemical treatments and biocontrol techniques are being implemented to preserve the nutritional value and safety of fresh produce [2]. Emerging non-thermal technologies are moving into the spotlight as sustainable alternatives to conventional heat-based methods, which are often highly water-consuming and can cause detrimental effects on quality aspects, such as nutrient and flavor losses [19,20,21,22]. Non-thermal technologies (Table 1), offer strong sustainability benefits, such as reduced energy use, preservation of fresh-like quality, and avoidance of chemical sanitizers. The most common challenges include high equipment costs, limited penetration depth, and scalability issues for industrial adoption. Some methods may also induce undesirable quality changes (e.g., oxidation, softening) if not properly optimized. Therefore, selecting the appropriate technology requires balancing process effectiveness, product characteristics, and economic feasibility, often leading to hybrid or combined approaches to achieve optimal results.

## 3. Innovations in Eco-Friendly Packaging and Regulatory Challenges

To contextualize these challenges, the following sections review technological options while critically evaluating limitations, scalability constraints, economic feasibility, and areas requiring further research effort.

Packaging plays a vital role in protecting perishable produce and extending shelf life, yet traditional plastic materials contribute to environmental pollution. Biodegradable polymers, compostable films, and edible packaging provide sustainable solutions without compromising protection. In addition, active and intelligent packaging systems can monitor freshness and enhance food safety.

Edible coatings, provide a sustainable approach by forming thin, edible layers composed of natural, biodegradable polymers, such as polysaccharides (chitosan, starch), proteins (whey protein, zein), and lipids (waxes) [10]. These coatings act as semi-permeable barriers that regulate gas exchange, reduce moisture loss, and suppress microbial growth. They offer antimicrobial activity and improve shelf life without residual odor or taste. Chitosan-based coatings, often enriched with essential oils or natural antioxidants derived from plant extracts (e.g., Aloe vera gel), are widely studied for enhancing the preservation of various fruits and vegetables [17]. Despite their advantages, edible coatings present several limitations, including variability in cost and challenges in achieving scalable, uniform application at the industrial level. Consumer acceptance may also be affected by concerns regarding coating visibility, texture, or perceived “added layers,” while companies must adapt processing lines and quality-control systems to ensure consistent performance. These factors highlight the need for careful optimization to balance efficacy, feasibility, and market acceptance.

The shift toward eco-friendly packaging is driven by the severe environmental issues associated with conventional petroleum-derived polymers, such as resource depletion and pollution [14]. Bioplastics, derived from renewable biomass resources like corn starch, sugarcane, or algae, offer a viable, often compostable and biodegradable alternative to traditional plastics. These materials, including polylactic acid (PLA) and starch-based films, break down naturally, aligning with the principles of a circular economy and reducing the carbon footprint [77]. Innovative packaging aims to improve quality and safety and extend shelf life with:

- Active packaging materials interact positively with the food or its environment to extend shelf life. This is achieved by incorporating bioactive compounds like antioxidants and antimicrobial agents, often sourced from agro-waste extracts, which are slowly released to the food surface [78].
- Intelligent packaging acts as an extension of the communication function, employing sensors and indicators (e.g., pH-sensitive dyes or bio-based sensors) to provide real-time, reliable information about the food’s condition, such as freshness, temperature, or spoilage status, directly to consumers [79].

New packaging technologies must be suitable for fresh produce quality maintenance while promoting environmental sustainability. Sustainable packaging designs focus on making solutions lighter and reusable, thereby reducing environmental impact and minimizing carbon footprints. Innovations include stackable containers and collapsible crates designed to enhance logistical efficiency and maintain produce integrity during transport [80]. Beyond these advances, recent literature highlights further developments and regulatory challenges shaping the transition toward sustainable packaging systems.

The food industry is rapidly shifting away from conventional petroleum-based plastics toward sustainable packaging solutions, particularly those involving biodegradable materials made from renewable biomass sources. Bioplastics, produced from renewable resources like corn starch, sugarcane, and algae, are key developments, often being compostable or biodegradable, with ongoing advances focusing on improving their durability and barrier properties [14]. Furthermore, renewable and biodegradable biocomposite materials—utilizing biomass sources such as coffee grounds and agricultural by-products like sugarcane bagasse and crop residues—are emerging as eco-friendly alternatives for disposable packaging [81,82]. Innovations like edible coatings are also crucial, as they help slow down ripening and extend shelf life during cold storage, directly supporting food quality preservation and waste reduction, just considering that in 2012, the global plastic production volume was 288 million tons [83].

From a regulatory perspective, innovation is hindered by the fragmented global framework for food-contact materials. Requirements differ widely between the EU, U.S., and emerging economies, generating uncertainty and compliance costs for manufacturers. The need for harmonized design certifications is increasingly recognized as essential to ensure global marketability. Consumer perception is another pivotal factor, especially in emerging markets where the concept of “eco-friendly packaging” is still poorly defined [13]. Most consumers associate sustainability primarily with biodegradability and recyclability, while affordability and aesthetic appeal remain decisive for market uptake.

In parallel, concerns over microplastic contamination—highlighted by EFSA in 2016—have accelerated global policy responses, including the EU ban on single-use plastics introduced in 2021 [84]. As shown by Khandeparkar et al. [14] traditional plastic packaging continues to cause environmental damage, raising the need for natural-source polymers as safer alternatives. Effective packaging materials must be cost-efficient, mechanically robust, and capable of providing excellent barrier protection, while simultaneously supporting innovations that lower carbon emissions, enhance food preservation, and ensure product safety [14].

However, the food industry faces a significant challenge in creating packaging that is both sustainable and affordable. Traditional packaging materials, primarily made from petroleum-based plastics, contribute to environmental pollution, resource depletion, chemical leaching into food, and difficulties in recycling. In response to growing environmental concerns, the industry is shifting toward sustainable packaging solutions, with a focus on biodegradable materials made from renewable biomass sources. A key development in this area is the rise of bioplastics—produced from renewable resources like corn starch, sugarcane, and algae—that are often compostable or biodegradable. Advances in bioplastics aim to improve their durability and barrier properties, making them more practical while addressing ecological and health issues. Currently, renewable and biodegradable biocomposite materials are gaining significant attention as eco-friendly solutions across various fields, including smart food packaging, biomedical applications, drug delivery, bio-membranes, automotive components, and industrial composting. Recent advancements in the fabrication methods and practical uses of biocomposites were explored by Moustafa et al. [81]. Biomass sources such as coffee grounds, nanocellulose, and date stones are being utilized as innovative reinforcing agents in biodegradable polymers to enhance their performance. A summary of he discussed materials is presented in Table 2.

Nevertheless, the use of lignocellulosic materials as reinforcements poses some challenges, such as high moisture absorption, low wettability, and poor compatibility with many biopolymers. To address these limitations, the review suggests new processing strategies aimed at producing high-performance lignocellulosic-reinforced materials with improved characteristics. Additionally, it discusses the eco-friendly modification of organoclay (OC) using natural antibacterial substances like rosin and stearic acid to create non-toxic, expanded OC. This modified OC can serve both as a compatibilizer and reinforcing agent for otherwise incompatible biopolymers such as chitosan, carboxymethyl cellulose (CMC), and polylactic acid (PLA). Moreover, are important outlines future directions and highlights environmental challenges, including CO_2_ emissions and the need for risk assessments related to the use of bionanomaterials [81]. Green technology has become increasingly important in the packaging industry, particularly through the use of renewable, bio-based materials derived from agricultural waste. This approach helps manage waste, reduce stubble burning, and conserve forest resources. With the global shift away from single-use plastics, lignocellulosic materials show strong potential for disposable packaging. However, challenges remain regarding their degradation, chemical safety, and commercial use. The recent advancements in transforming agricultural by-products like sugarcane bagasse and crop residues into sustainable packaging solutions are highlighted by Bhardwaj et al. [82].

The large-scale adoption of bioplastics is primarily limited by high production costs and the difficulty of achieving competitive cost–performance ratios compared with petroleum-based plastics. PLA and PHA, in particular, remain expensive due to costly substrates and processing requirements, although blending strategies and the use of low-cost agro-waste materials (e.g., chitosan, cassava starch, citrus pectin) can partially mitigate expenses.

Scalability also poses major constraints, as most bioplastic systems are still developed at laboratory scale, and industrial implementation is hindered by challenges in feedstock collection, storage, transport, and the lack of efficient recycling and recovery infrastructure. Regulatory acceptance further complicates commercialization: strict guidelines governing food-contact materials, nanomaterial migration, and toxicity—especially within the EU—add complexity and increase compliance costs. Adoption is additionally impeded by performance limitations, including low mechanical strength, poor thermal stability, and high moisture sensitivity of many protein-, starch-, and cellulose-based materials. Consumer price sensitivity and inadequate post-use management systems further restrict market uptake, underscoring the need for improved material performance, cost-effective production, and supportive policy frameworks.

A broader view of biocomposites reveals expanding applications beyond food packaging—including biomedical, automotive, and industrial sectors—but their safe deployment requires further evaluation of CO_2_ emissions, toxicity, and life-cycle impacts.

Table 3 highlights the increasing academic interest in these topics, including the recent establishment of specialized journals such as Sustainable Food Technology (2024), which provide dedicated academic platforms for research dissemination.

## 4. The Circular Bioeconomy as an Integrated Approach to Waste and Bioactive Compound Valorization

Advancing sustainable postharvest solutions increasingly requires an integrated circular-bioeconomy perspective, in which innovative processing strategies extend beyond packaging and operate together with waste valorization to enhance resource efficiency and minimize environmental impact. Emerging techniques such as ultrasound exemplify this approach, as they aim to optimize extraction and preservation while improving process efficiency [87]. Meanwhile Fu et al. provide a broad overview of sustainable processing research, distinguishing between end-of-pipe and clean technologies and aligning the latter with the UNEP definition of clean production as a preventative, efficiency-oriented environmental strategy [88]. Their systematic review screened 964 articles but identified only 34 that met strict criteria, ultimately classifying sustainable technologies into CO_2_/emission reduction, material/fuel substitution, energy/material efficiency, and recycling. They also highlight key drivers of adoption, including regulatory pressure, firm characteristics, technological capability, certified systems, and inter-organizational cooperation [88].

Food system resilience increasingly depends on the ability to transform waste streams into valuable resources. Globally, one-third of food production is lost or wasted each year, encouraging the United Nations to set the target of halving food waste by 2030.

In this context, Mady et al. conducted a systematic review on food by-products from eggplant, taro, okra, and coconut, demonstrating their richness in phenolics (anthocyanins, luteolin, quercetin, catechins) and chlorophyll—compounds with antioxidant, anti-inflammatory, anti-mutagenic, and anti-genotoxic properties. These findings highlight the potential for developing new functional ingredients and nutraceuticals, though more phytochemical, biological, industrial, and clinical research is needed to enable full-scale applications [89].

## 5. Advanced Processing and New Evaluation Paradigms

Starting from the opportunities offered by the improved use of bioactive compounds derived from food by-products, the discussion naturally extends to the role of innovative processing and valorization strategies in shaping more sustainable and healthier food systems. Within this context, Ultra-Processed Foods (UPFs) represent a particularly debated category: although they are often criticized for their association with adverse health outcomes such as obesity and cardiometabolic risk, recent attempts to incorporate nutrient-rich residues or bioactive compound–containing by-products into UPF formulations add further complexity. Yet, the extent to which these processing approaches influence health effects—and how they are perceived by consumers—remains unclear. As highlighted in the review by Capozzi et al. [5] the authors aimed to reassess existing evidence, identify research gaps, and examine the potential of advanced processing technologies to enhance food matrices, valorize by-products, and promote sustainability within a circular economy framework. The authors advocate for new paradigms of food evaluation—anchored in enginomics, signaling, and precision nutrition—which must be supported by digital and AI tools, emerging technologies, multidisciplinary research, and collaboration between academia and industry.

In continuity with these emerging frameworks, the attention shifts entirely to the circular bioeconomy. This approach is positioned as the central, holistic, and sustainable strategy for food processing and transformation [90]. The framework focuses on the revalorization of agro-food by-products through conventional and advanced extraction strategies to create functional, health-promoting foods and beverages. These practices are framed as holistic and sustainable, balancing economic and social development with environmental protection. Effective implementation requires not only technological innovation but also supportive government policies and legal frameworks. Furthermore, consumer choices play a decisive role in shaping environmental, social, and economic impacts, reflecting current trends that show a growing demand for eco-friendly, minimally processed products with low carbon footprints and freedom from genetically modified organisms.

### 5.1. Comparative Analysis of Sustainable Food Technologies

In order to advance these circular and health-oriented perspectives, the global food industry is increasingly compelled to adopt innovative postharvest solutions and processing technologies that reduce waste and environmental burdens. Assessing these approaches in terms of efficiency, cost, and scalability (as summarized in Table 4) highlights the shift required from conventional methods to more sustainable alternatives, while also revealing important research gaps that remain to be addressed.

### 5.2. Highlighting Research Gaps and Challenges

As reported in Table 4, significant gaps still remain in research on the adoption and implementation of sustainable technologies, particularly regarding widespread scale-up and economic feasibility, according to the main points summarized below:

- Scalability and Standardization: Despite its promise, ultrasound technology has limited reports at pilot or commercial scale. Key challenges include the lack of standardized methodologies, operational parameters, and data on thermophysical properties (e.g., density, heat capacity), making scale-up predictions unreliable. Research on the design, sizing, and modeling of ultrasound units, especially when integrated with other green technologies, is also scarce [87].
- Cost Prediction and Economic Viability: More studies are needed to assess the economic feasibility of green processing methods like ultrasound. Policymakers and technology providers require clearer information on relative advantages and costs to identify which technologies are suitable for promotion [88].
- Material Gaps in Packaging: Renewable packaging materials, such as lignocellulosic composites, face limitations in degradation behavior, migration properties, and commercial potential. Many biopolymer films exhibit low tensile strength, poor thermal stability, and low moisture resistance, constraining commercialization [14].
- Processing and Health Links: The direct role of specific processing techniques in linking Ultra-Processed Foods (UPFs) to adverse health outcomes remains unclear. Research is needed to refine or develop food evaluation and classification systems that incorporate processing factors impacting health and wellness [5].

Research should focus on underexplored process technologies, particularly material/fuel substitution and recycling approaches, while simultaneously addressing gaps through standardized methodologies, improved economic models, and targeted studies on material science and industrial scaling [87,88].

## 6. Conclusions and Future Perspective

The global food sector is undergoing a substantive paradigm transformation, prompted by the imperative to reduce postharvest losses—reaching up to 40% in fresh produce—and to mitigate the associated environmental burden. These pressures intersect with overarching global risks, notably those related to climate change mitigation and adaptation, thereby underscoring the urgency of transitioning toward more sustainable and resilient agri-food systems. Sustainable postharvest management increasingly relies on the deployment of advanced processing technologies, intelligent and energy-efficient storage systems, and environmentally compatible packaging solutions. Of particular relevance is the accelerating shift from conventional petroleum-based plastics toward biodegradable biopolymers and bio-composites derived from renewable biomass, supported by growing evidence of their environmental and functional advantages. Nonetheless, substantial impediments remain, including elevated production costs, limited scalability, regulatory inconsistencies across jurisdictions, and variable levels of consumer acceptance. These constraints must be addressed to enable broader industrial integration.

Achieving long-term sustainability necessitates a comprehensive and integrated strategy grounded in the principles of the circular bioeconomy. This involves not only the advancement of innovative technologies, but also the systematic valorization of agri-food by-products. Concurrently, emerging materials and techniques must conform to food-contact regulatory frameworks and remain responsive to evolving consumer expectations for environmentally responsible solutions. To facilitate the translation of these innovations into scalable, high-impact applications, future efforts should prioritize:

- Harmonized international regulatory frameworks for bioplastics and biodegradable packaging;
- Investment in infrastructure and technological capacity, particularly within low- and middle-income regions;
- Strengthened interdisciplinary collaboration spanning engineering, nutrition, microbiology, policy, and data science;
- Development of advanced decision-support systems integrating life-cycle assessment, artificial intelligence, and precision nutrition.

By addressing these strategic priorities, sustainable postharvest innovations can make a substantive contribution to achieving SDG 12.3, reinforcing global food system resilience, and advancing environmental stewardship.

## Figures and Tables

**Table 1:** Non-Thermal Sustainable Processing Technologies.

Technology	Mechanism and Applications	Limitations/Challenges	Citations
**High Hydrostatic Pressure (HHP)/High Pressure Processing (HPP)**	A non-thermal process used to achieve microbial inactivation and enzyme denaturation. Applied to minimally processed horticultural commodities and whole produce. HHP can stimulate the accumulation of nutraceutical compounds.	Very high equipment cost; limited applicability for porous or gas-containing foods; potential changes in texture; scalability depends on batch systems.	[2,5,14,23,24,25,26,27,28,29]
**Pulsed Electric Fields (PEF)**	Uses short, high-intensity electric field pulses to ensure safe food with minimal heat production. Effective in reducing enzymatic activity and improving quality attributes. PEF can reduce browning index and acrylamide content in ready-to-eat foods.	High initial investment; only suitable for pumpable/liquid foods or homogeneous matrices; potential electrode corrosion; complex process optimization.	[30,31,32,33,34,35,36,37]
**Cold Plasma**	Non-thermal decontamination technology that produces reactive oxygen and nitrogen species (RONS) through gas ionization to eliminate surface microbes. Increasingly used as a replacement for conventional sanitation treatments because it preserves product quality.	Limited penetration depth (surface-only treatment); potential oxidation effects on sensitive compounds; equipment standardization still lacking; regulatory variability.	[38,39,40,41,42,43,44,45,46,47,48,49,50,51]
**Ultraviolet-C (UV-C)**	Postharvest management tool acting through microbial DNA disruption, induction of host defense (e.g., phytoalexin synthesis), and delayed ripening via interference with ethylene signaling.	Ineffective on shaded/irregular surfaces; risk of excessive tissue stress; requires safety precautions; dose uniformity can be challenging.	[52,53,54,55,56,57]
**Pulsed Light (PL)**	Non-thermal, non-chemical method that uses brief bursts of intense broad-spectrum light to inactivate surface microorganisms through photochemical and photothermal effects.	Limited penetration; potential surface heating or discoloration; strict safety requirements for operators; equipment costs moderate to high.	[58,59,60]
**Ozone + Modified Atmosphere Packaging (MAP)**	Ozone exposure combined with MAP can significantly extend the postharvest storage life of small berries by reducing microorganisms and delaying spoilage.	Residual ozone must be controlled to avoid off-flavors; regulatory limits vary by region; sensitive commodities may suffer oxidative damage.	[4,61,62,63,64,65]
**Ultrasound Technology**	Recognized as a green technique for food preservation. Effective for microbial inactivation and enzyme denaturation, improving quality while reducing reliance on thermal treatments.	Can cause tissue softening or cell damage at high intensities; limited penetration; energy efficiency depends on medium and equipment design.	[66,67,68,69,70,71,72,73,74,75,76]

**Table 2:** Comparison of biodegradable packaging materials (bioplastics).

Bioplastic	Origin/Source	Key Characteristics & Functionality	Barrier Properties	Biodegradability	Cost/Economic Factors	References
**Polylactic Acid (PLA)**	Derived from renewable resources (e.g., microbial fermentation of lactic acid from corn or sugar beets).	Biodegradable and bioactive polymer. Exhibits transparency. Offers high modulus and strength. Has good mechanical performance. Is thermoplastic and easily processable. Inherently brittle, limiting industrial use, but properties can be modified by adding fillers like starch.	Possesses excellent barrier properties. Good gas barrier properties.	Biodegradable. Degradable by the action of microorganisms under appropriate conditions. Recyclable back to lactic acid.	High cost, which significantly constrains commercial application. Blending with other biopolymers is a viable solution to lower costs.	[14,77]
**Starch**	Renewable, environmentally-friendly, and inexpensive. Extracted from agricultural products such as corn, cassava, and potato.	Highly available. Used as a filler to modify PLA properties. Characterized by its hydrophilic nature. Can be modified into starch nanocrystals (S-NCs) to improve barrier and physicochemical characteristics of bio-composites. Forms edible and biodegradable films.	Provides high mechanical strength and water-gas barrier. Generally, has a low water vapor barrier.	Biodegradable/Compostable. Biodegradability of PLA-blends increases as the starch content increases.	Inexpensive material. Low material cost when blended with chitosan.	[14,16,77,85,86]
**Chitosan (CS)**	A polysaccharide mainly obtained by the partial deacetylation of chitin. Primarily a byproduct of crustacean, fish, and seafood processing waste.	Biocompatible, non-toxic, and abundant. Cationic nature gives it strong antimicrobial activity against bacteria, fungi, and yeast. Possesses excellent film-forming properties, toughness, flexibility, and durability. Used extensively for edible coatings and films.	Forms outstanding barriers against oxygen and carbon dioxide. Generally, lacks resistance to moisture and has a low water vapor barrier.	Biodegradable. Some biopolymers like it can degrade in just a few weeks.	Low cost and readily available in nature.	[14,16]
**Pectin**	Complex anionic polysaccharide. Extracted from citrus peels (lemon, orange, grapefruit), apple pomace, cocoa husk, etc. Grapefruit peel pectin (GFPec)	Remarkable gelling, stabilizing, and thickening edible agent. Generally regarded as safe, multifunctional natural food additive (E440). GFPec is classified as high-methoxyl (HM) and high-ester, having strong film-forming capacity.	Good oxygen barrier. Active films of GFPec enriched with medium-density polyethylene (MD-LPE) show the ability to protect light-sensitive foods. Water Vapor Permeability (WVP) of neat GFPec film was higher than commercial pectin.	Biodegradable. Considered a desirable alternative to synthetic petrochemical-derived materials.	Cost-effective when mobilized from citrus peel wastes.	[78,82]
**Polyhydroxyalkanoates (PHA)**	A type of polyester produced naturally by bacteria (e.g., Bacillus megaterium). Produced in environments subjected to more carbon and nutrient limitation.	Biocompatibility and non-toxic qualities. Shows versatility in mechanical characteristics. Has a strong hydrophobic nature and thermoplasticity. Great potential to replace traditional plastics in food packaging.	High water vapor barrier. Strong barrier qualities against CO_2_ and oxygen.	Biodegradable.	High cost of initial supplies is a fundamental limitation in large-scale manufacture.	[14]
**Alginate**	Naturally derived anionic polymer from brown seaweed (marine macroalga).	Versatile biopolymer. Non-toxic, biocompatible, and inexpensive hydrocolloid. Widely used as a thickening agent, for gel formation, and as a colloidal stabilizing agent in beverages. Can form strong films.	Good barrier properties against lipids and gases. Inferior barrier against water vapor.	Fully biodegradable.	Low-cost.	[14,16]
**Gelatin**	A protein compound obtained from animal cartilage, bones, skin, and fish or seafood processing wastes.	Biocompatibility. Ability to form a transparent gel. Hydrophilic biopolymer showing good affinity and compatibility with chitosan. Protein-based films typically have low tensile strength and low heat endurance.	Provides good protection from oxygen and aromatic compounds.	Biodegradable.	Less expensive.	[14,16]
**Cellulose**	Abundantly found in plants and bacteria. Sources include lignocellulosic agricultural waste (e.g., rice straw, sugarcane bagasse, corn cob).	Biocompatibility and non-toxic. Cellulose nanocrystals (NCC/CNW) exhibit great tensile strength and high stiffness. Used in films, coatings, and bioplastics.	Offers low permeability to oxygen. Has a hydrophilic nature, resulting in low water vapor barrier.	Biodegradable. Used in the development of biodegradable and home compostable configurations.	Low density and relatively lower cost (when using lignocellulosic agricultural biomass).	[14,81,82,86]

**Table 3:** Specialized Journals in the field grouped by topics, launch dates and research priorities.

Topic	Main Journal(s)	Start Year	Key Focus Areas
Sustainable processing technologies	*Journal of Cleaner Production;* *Sustainability*	1993; 2009	Sustainable industrial processes, food processing technologies, green innovation
Eco-friendly packaging materials	*Sustainable Food Technology; Polymers*	2024; 2009	Biodegradable packaging, bio-based materials, active and smart packaging
Smart storage systems	*Sustainable Food Technology*, *arXiv (preprints); Foods*	2024; 2025; 2012	Intelligent packaging, sensors, real-time monitoring of food shelf-life
Postharvest management for food waste	*Sustainable Food Technology*, *Journal of Horticultural Science and Biotechnology; Foods*	2024; 2022; 2012	Postharvest preservation, waste reduction, nanotechnology in storage
Sustainable cold chain logistics	*Journal of Cleaner Production*	1993	Environmentally friendly supply chains and refrigerated logistics
Nanotechnology in postharvest applications	*Journal of Horticultural Science and Biotechnology*, *Sustainable Food Technology; Applied Sciences; Polymers*	2022; 2024; 2011; 2009	Nanocoatings, nanosensors, smart packaging, shelf-life extension
Integrated postharvest solutions	*Sustainable Food Technology*, *Journal of Cleaner Production*	2024/1993	Combined solutions: processing, packaging, storage, logistics

**Table 4:** Comparative Analysis of Sustainable Technology Options.

Technology	Efficiency & Performance	Cost & Affordability	Scalability & Implementation
**Conventional Processing (e.g., thermal treatment, traditional extraction, petroleum plastics)**	Often compromises food quality due to high temperatures. Traditional drying is very energy-intensive and time-consuming. Extraction often requires high quantities of organic solvents and prolonged processing times [87]	Frequently cost-effective initially (e.g., manufacturing petroleum-based plastics) [14]	Established and widely available, but environmentally detrimental [14,87]
**Ultrasound Technology (Green Processing)**	High efficiency: Superior to conventional methods, offering reduced processing time, higher extraction yields, and reduced solvent consumption. High ultrasonic power effectively enhances bioactive compounds and achieves microbial inactivation. Can shorten drying time when used as pre-treatment [87].	While green techniques are generally expensive, the advantages (e.g., food safety, higher nutrition, shorter times) often outweigh the expense. Considered a cost-effective and rapidly evolving technique that ensures quality retention while lowering energy consumption [87].	Found to be a rapidly evolving and scalable technique, suggesting potential for industrial use. Optimization studies indicate specific parameters (e.g., medium power for extraction, higher power with mild heat for pasteurization) that could guide industrial scaling [87].
**Bioplastics Biocomposites**	Offers favorable mechanical properties, transparency, and high barrier properties compared to other biopolymers (e.g., PLA). Innovations enhance performance in areas like mechanical strength, barrier properties, and antimicrobial activity [14].	Raw materials and processes are generally more expensive than petroleum-based plastics. The high cost of materials like PLA constrains application, although blending with other polymers can reduce costs [14].	Commercialization is limited by the large initial capital required. Challenges exist in scaling up due to the need for compatibility between materials (e.g., lignocellulosic materials often struggle with high moisture absorption and poor compatibility with biopolymers) [14].

## Data Availability

No new data were created or analyzed in this study. Data sharing is not applicable to this article.

## Abbreviations

The following abbreviations are used in this manuscript:

UPFs	Ultra-Processed Foods
FLW	food loss and waste
SDGs	Sustainable Development Goals
FAO	Food and Agriculture Organization (of the United Nations)
EU	European Union
EFSA	European Food Safety Authority
PGR	plant growth regulators
HHP	High Hydrostatic Pressure
PEF	Pulsed Electric Fields
UV	ultraviolet
OC	organoclay
CMC	carboxymethyl cellulose
PLA	polylactic acid
CO_2_	carbon dioxide
PHA	Polyhydroxyalkanoates
GFPec	Grapefruit peel pectin
MD-LPE	medium-density poly-ethylene
S-NCs	Starch NanoCrystals
NCC	Nanocrystalline Cellulose
CNW	Cellulose Nanowhiskers
HM	high-methoxyl
WVP	Water Vapor Permeability
UNEP	United Nations Environment Program
